# The tree snail on Rota Island, Northern Mariana Islands, long identified as *Partula gibba* (Partulidae), is a different species

**DOI:** 10.3897/zookeys.1037.56303

**Published:** 2021-05-17

**Authors:** David R. Sischo, Michael G. Hadfield

**Affiliations:** 1 Kewalo Marine Laboratory, Pacific Biosciences Research Center, University of Hawai‘i at Mānoa, 41 Ahui St., Honolulu, 96813 Hawai‘i, USA University of Hawai‘i at Mānoa Honolulu United States of America; 2 Department of Land and Natural Resources, Division of Forestry and Wildlife, State of Hawai‘i, 1151 Punchbowl St. Rm. 325, Honolulu, 96813 Hawai‘i, USA Department of Land and Natural Resources, Division of Forestry and Wildlife Honolulu United States of America

**Keywords:** Cryptic species, Mariana Islands, Micronesia, Mollusca, narrow-range endemic, systematics

## Abstract

Tree snails in the family Partulidae are widespread across the tropical Pacific, with endemic species occurring on most high islands. Partulid species have faced catastrophic range reductions and extinctions due primarily to introduced predators. Consequently, most extant species are threatened with imminent extinction. The U.S. administered Mariana Islands, consisting of Guam in the South and the Commonwealth of the Northern Mariana Islands (CNMI) in the north, historically harbored six endemic partulid species, half of which are thought to be extinct. While conducting a phylogenetic assessment of *Partula gibba*, an extant tree-snail with a range spanning at least seven islands within the archipelago, it was discovered that what has been identified as *P. gibba* on the island of Rota is a misidentified cryptic species. Here we use molecular phylogenetics, shell morphometrics and reproductive anatomy to describe it as a new species, *Partula lutaensis***sp. nov.**. Because the new species has suffered population declines and has a restricted range, consisting solely of the small island of Rota, we highlight the urgent need for conservation measures.

## Introduction

**Figure 1.**
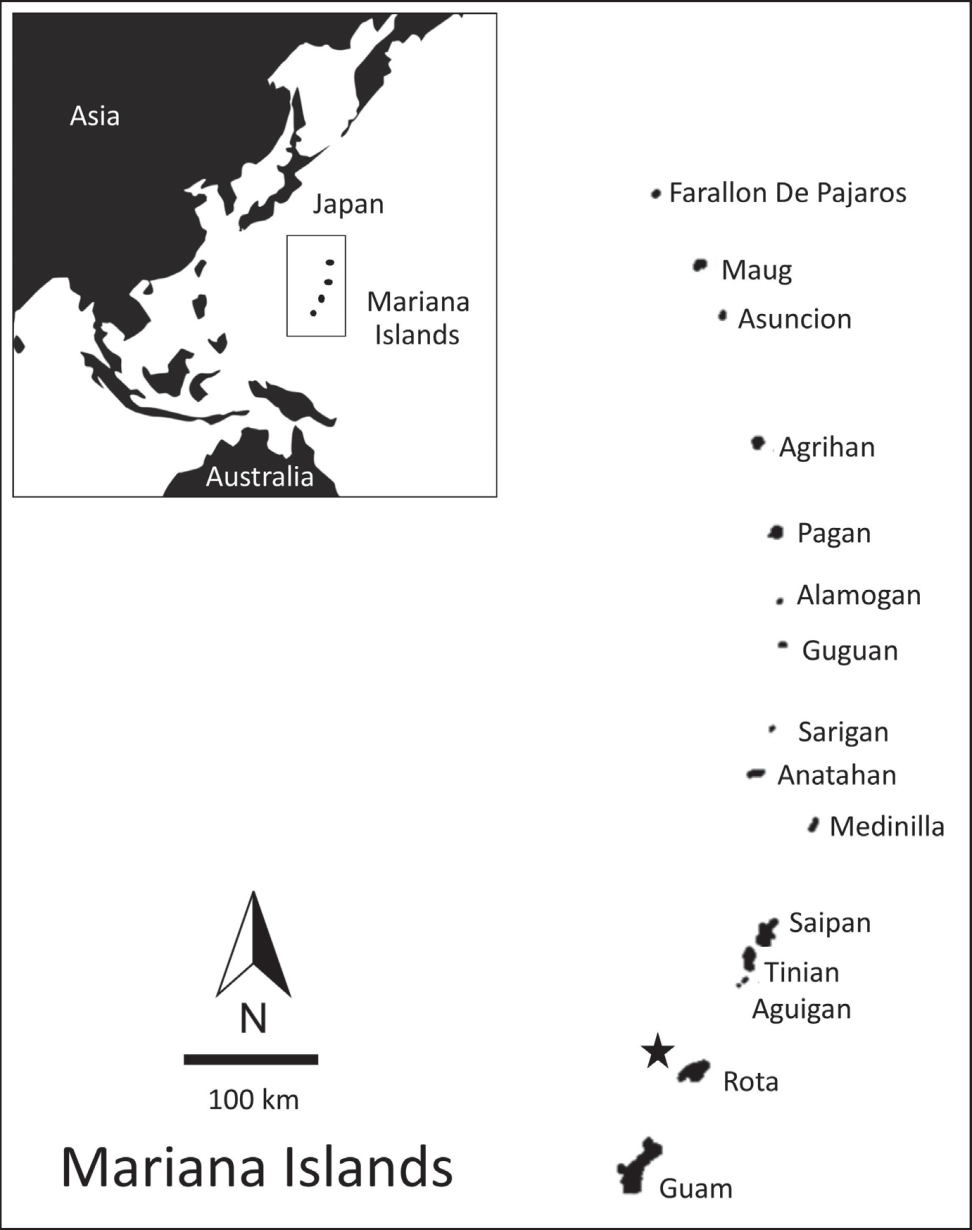
Map of the Mariana Archipelago with a star indicating the location of Rota Island.

The tree-snail family Partulidae is known from islands across the tropical Pacific (Cowie 1992). While most of the known diversity occurs in the eastern portion of the family’s range, particularly the Society Islands for the genus *Partula* and the Samoan Islands for the genus *Samoana*, the progenitors of both genera likely came from further west (Lee et al. 2014). The 15 islands of the Mariana Archipelago in the western Pacific (Fig. 1) historically harbored six described species. Two of them, *Partula radiolata* (Pfeiffer, 1846), and *Partula salifana* Crampton, 1925, were known only from the island of Guam, while *Partula desolata* Bauman & Kerr, 2013, described from sub-fossil shells, and *Partula langfordi* Kondo, 1970, were described as single-island endemics from Rota and Aguigan, respectively. The only two Mariana species with multi-island distributions are *Samoana fragilis* (Férussac, 1821), known from both Guam and Rota, and *Partula gibba* Férussac, 1821, known from seven islands, from Guam, in the south, to Pagan Island in the north. In a recent publication, we described the discovery of a cryptic species of *Partula* on the island of Rota, based on molecular evidence (Sischo and Hadfield 2017). Due to greatly similar shell shape, this species had been identified as *Partula gibba* in prior surveys and publications (Kondo 1970; Bauman 1996; Bauman and Kerr 2013; Hadfield 2015). Adding further taxonomic confusion, a colony of *Partula* once maintained at the Invertebrate Conservation Center, Zoological Society of London in London, was labeled *Partula langfordi*, although it was originally collected on Rota (Goodacre and Wade 2001; Sischo and Hadfield 2017). Because *P. langfordi* was described as endemic to the island of Aguigan, snails bearing that name in the London collection were likely not *P. langfordi* (Kondo 1970). Unfortunately, *P. langfordi* is now thought to be extinct (Smith 2013; J. Liske-Clark, Northern Mariana Department of Fish and Wildlife, personal communication). The name of Aguigan Island is variously spelled on different maps and in different resources, including: Agiguan, Agijuan and Aguijan. We apply here the spelling currently in use by CNMI bureaus, the NOAA and elected officials in the CNMI.

To distinguish the new Rota *Partula* species from other extant species in the Mariana Archipelago, we paired our previously published phylogeny (Sischo and Hadfield 2017) with anatomy of the male reproductive tract, the latter having been used extensively as a diagnostic trait for *Partula* species (Pilsbry 1909; Pilsbry and Cooke 1934; Kondo 1955, 1968, 1970; Gerlach 2016; Slapcinsky and Kraus 2016). Because we were not able to extract useful DNA from preserved tissue of *Partula langfordi*, we were unable to carry out molecular phylogenetics with that species. In addition to anatomy of the male reproductive tract, we further distinguished *P. langfordi* from the species on Rota by replicating the shell morphometric analysis originally conducted by Kondo (1970) in his study of *P. langfordi* and co-occurring *Partula gibba* on the island of Aguigan. Here we describe *Partula lutaensis* sp. nov. and designate type material.

## Material and methods

Due to the endangered status of all partulid species from Guam and the Commonwealth of the Northern Mariana Islands, we have elected to not include specific location information of any of the extant colonies or type specimen material in this paper. However, this information has been deposited in the Bernice Pauahi Bishop Museum with each specimen.

### Shell morphological assessment

**Figure 2.**
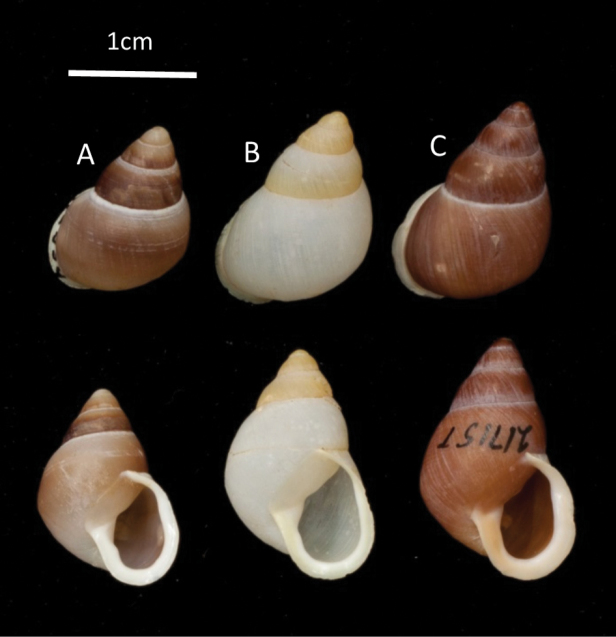
A typical shell of *P. langfordi* from Aguigan (**A**) compared to light and dark shell morphs of *P. lutaensis* sp. nov. from Rota (**B** and **C**). The three shells are shown in abapertural view in the upper row and apertural view below.

Kondo (1970) found no differences in conchology, aside from non-overlapping shell sizes, between *P. langfordi* and sympatric *P. gibba* on the island of Aguigan. Because *Partula* from Rota had been mistakenly identified as *P. langfordi*, we carried out a similar comparison. Shells of *P. langfordi* from the mollusc collection housed at the Bernice Pauahi Bishop Museum (**BPBM**) in Honolulu, Hawai‘i were used for analysis. BPBM lot numbers 213092, 21309, 213104, 213012, 213024, originally collected by Y. Kondo from Aguigan Island were compared with shells from what was then labeled as *P. gibba* (BPBM lot numbers, 217155, 213251, 213248, 213151, 213241) originally collected on Rota (Fig. 2). Lengths and widths were measured to 0.01 mm from 48 shells of *P. langfordi* and 47 shells of *P. gibba* with precision calipers. Shell length was measured parallel to the shell axis from the apex to the base of the aperture, and width was measured perpendicular to the shell axis across the widest portion of the shell. Means (M) and standard deviations (s.d.) are reported. Adult *Partula* spp. stop growing and become sexually mature when they form a characteristic thickened flare around the aperture of their shells, here referred to as a lip. All shells measured were lipped, indicating they were mature adults (Cowie 1992). Mean shell length and width were compared with independent-samples, two-sided t-tests, assuming unequal variances, using Microsoft Excel (version 16.44). Extensive shell metrics and comparisons for all partulid species of the Mariana Islands are found in historical publications (Crampton 1925; Kondo 1955, 1970). More recently Gerlach (2016) carried out extensive morphometric analyses of partulid shells, including *P. lutaensis* sp. nov., there referred to as *Partula* sp. (Rota). We therefore have made no other shell size comparisons. Lacking phylogenetic data for *P. langfordi*, our objective here was to replicate Kondo’s shell size comparison between *P. langfordi* and sympatric *P. gibba* to determine whether *P. langfordi* and *P. lutaensis* sp. nov. are similarly distinct.

### Morphology of the male reproductive tract

Many taxonomic descriptions of partulid snails have emphasized reproductive anatomy, particularly differences in the male part of the reproductive tract, to differentiate species (Pilsbry 1909; Pilsbry and Cooke 1934; Kondo 1955, 1968; Gerlach 2016; Slapcinsky and Kraus 2016). For this purpose, we obtained preserved specimens of *Partula radiolata* (lot nos. 21462 [2] and 213605 [1]) and *P. gibba* (lot nos. 214256 [2] and 214179 [1]) from Guam and those recorded as *P. gibba* from Rota (lot nos. 188958 [2], 213131 [2], 213152 [3]) from the extensive collections of the BPBM in Honolulu, Hawai‘i. In selecting preserved museum specimens from Rota, we endeavored to obtain snails from the same or very near sites where snail tissue samples were collected for DNA analysis. Kondo and others who collected at these sites separated ‘soft parts’ from many shells for inclusion in the Bishop Museum collections and maintained the same lot numbers for the shells and preserved bodies for snails collected at one site at the same time. This allowed us to examine the shells before carrying out the dissections to make certain that the shells matched the shells of the snails from which we had collected small tissue samples for DNA analyses. In all cases, we were successful in this matching. Kondo (1970) found no difference in the male reproductive tracts of *P. langfordi* and *P. gibba*. We therefore did not dissect specimens of *P. langfordi*.

The following specimens were dissected.

- Partula radiolata (Pfeiffer, 1846) from Guam: BPBM no. 214262, 2 spms; BPBM no. 213605, 1 spm.
- Partula gibba Férussac, 1821 from Guam: BPBM no. 214256, 2 spms; 214179, 1 spm.
- Partula sp. nov. from Rota, as P. gibba in BPBM collections: BPBM no. 213151, 3 spms; 188958, 1 spm.; 213132, 2 spms; 213128, 1 spm.

The museum specimens were stored in 90% ethanol. Before dissecting them, we transferred them to three changes of fresh water and carried out the dissections under water. The reproductive tracts of the snails were exposed by cutting the right-dorsal wall of the snail with a fine scalpel. Then, using fine forceps, the reproductive tract was carefully exposed and the ducts teased apart. Dissections were photographed with a Canon camera mounted on a Zeiss dissection microscope. Outline drawings were made by tracing duct contours from photos using Adobe Illustrator.

### DNA analysis

During our collecting trip to Rota in 2010 only small tissue samples were collected for DNA analysis. Following the discovery of a cryptic species on Rota described in Sischo and Hadfield (2017), we were sent five newly collected voucher specimens from Rota by the CNMI Division of Fish and Wildlife to serve as type material for this new cryptic species. Unfortunately, our attempts to extract DNA from these ethanol-preserved specimens failed for unknown reasons. In the interim, all *Partula* species from Guam and the Commonwealth of the Northern Mariana Islands were listed as Endangered under the U.S. Endangered Species Act (US Fish and Wildlife Service 2015). Due to the rarity of the new species and its listing status, we have been unable to obtain another full voucher specimen. To move forward with describing this species, three non-lethal tissue samples were collected from individuals of the same population as the original shell vouchers provided by the CNMI Division of Fish and Wildlife, as well as three samples from a new site not visited by Sischo and Hadfield (2017). Non-lethal tissue samples were collected following the methodology of Thacker and Hadfield (2000), originally developed for sampling Hawaiian tree snails. These tissue vouchers were used to confirm that the shell vouchers are *P. lutaensis* and not *P. gibba*. Tissue sample collection, tissue preservation, total cell DNA extraction, CO1 DNA amplification and CO1 phylogenetic analyses were carried out using the methods described by Sischo and Hadfield (2017).

## Results

### Systematics

#### Class Gastropoda Cuvier, 1795

**Subclass Heterobranchia Burmeister, 1837**

**Order Stylommatophora A. Schmidt, 1855**

**Superfamily Pupilloidea W. Turton, 1831**

#### Family Partulidae Pilsbry, 1900

##### Genus Partula

Taxon classificationAnimaliaStylommatophoraPartulidae

Férussac, 1821

E06C6DAC-A210-597B-85C4-BA1EBC10A774

###### Type species.

*Helix faba* Gmelin, 1791.

##### Partula lutaensis sp. nov.

Taxon classificationAnimaliaStylommatophoraPartulidae

6D583946-B900-5747-9D87-A83059996E69

http://zoobank.org/65D4AC75-488B-4A29-9773-5E0F5FB25449

###### Type material.

**Figure 3.**
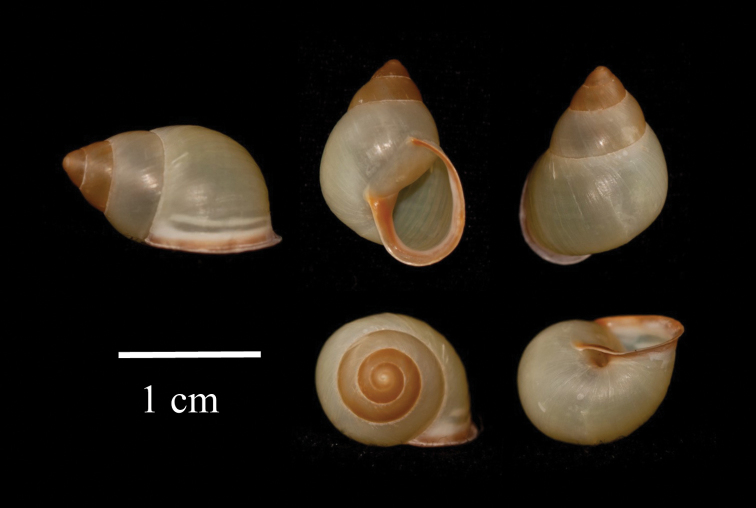
Shell holotype of *P. lutaensis* sp. nov.

***Holotype*.** Bishop Museum BPBM 284888 Fig. 3. Entire specimen collected by Jill Liske-Clark, 20/11/2014 from type locality. ***Paratypes*.**BPBM 284889, 2 specimens collected by Jill Liske-Clark, 20/11/2014, from type locality, and BPBM 284890, 3 specimens collected by Jill Liske-Clark, same date, from a second location on Rota Island, Commonwealth of the Northern Mariana Islands.

###### Type locality.

Rota Island, Commonwealth of the Northern Mariana Islands (CNMI).

###### Diagnosis.

**Figure 4.**
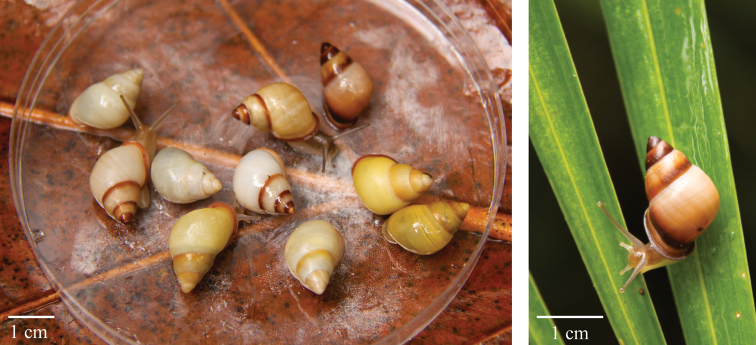
Left – Color morphs of *Partula lutaensis* sp. nov. found within a 10 × 10-meter quadrat. Right – Closeup of a *Partula lutaensis* sp. nov. with a dark shell.

***Shell*.** Shell dextral, moderately thin, ovate-conic, slightly perforate; umbilicus open; whorls moderately convex, suture adpressed; aperture ovate-elongate, slightly oblique; outer lip reflexed, thick, glossy; parietal lip glossy with light or dark coloration; color of embryonic whorls and post-embryonic whorls variable from shades of brown, buff, white and yellow with prominent white subsutural band; Measurements (*N* = 48 specimens from five lots in BPBM collections from Rota): height (= length) 15.98 mm, s.d. 0.75 mm; width, 10.64 mm, s.d., 0.24 mm. See Figure 4 for examples of shell color variation. Shell greatly resembles those of *Partula gibba* on Guam and Saipan (Crampton 1925).

###### Distinguishing shells of *P. lutaensis* from those of *P. langfordi*.

**Figure 5.**
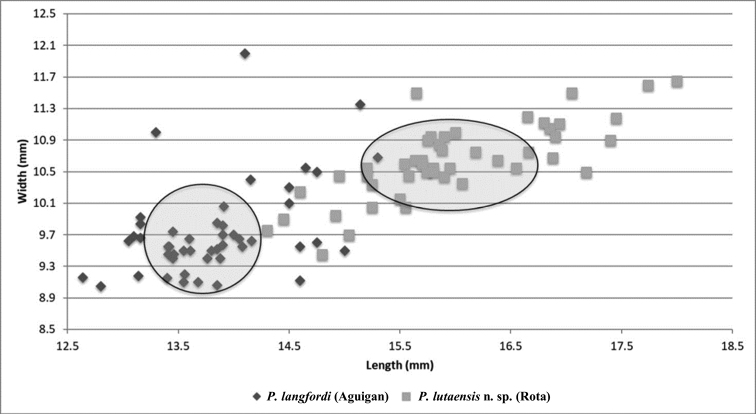
Scattergram of Bishop Museum shell measurements comparing shell width (y-axis) by shell length (x-axis) of 48 *P. langfordi* from Aguigan and 47 *P. lutaensis* sp. nov. from Rota. The mean shell length and width plus standard deviation of each species are encircled. All shells were lipped indicating snails were mature and had reached terminal growth.

Shell length of *P. langfordi* (*M* = 13.83 mm, s.d. = 0.37 mm, *N* = 47) was significantly shorter than the length of *P. lutaensis* sp. nov. (*M* = 15.98 mm, s.d. = 0.75 mm, *N* = 48), t(82) = -13.91, P < 0.001. Similarly, shell width of *P. langfordi* (*M* = 9.74 mm, s.d = 0.36 mm) was significantly less than that of *P. lutaensis* sp. nov. (*M* = 10.64 mm, s.d = 0.24 mm), t(90) = -8.02, P < 0.001 (Fig. 5).

###### Male reproductive system.

**Figure 6.**
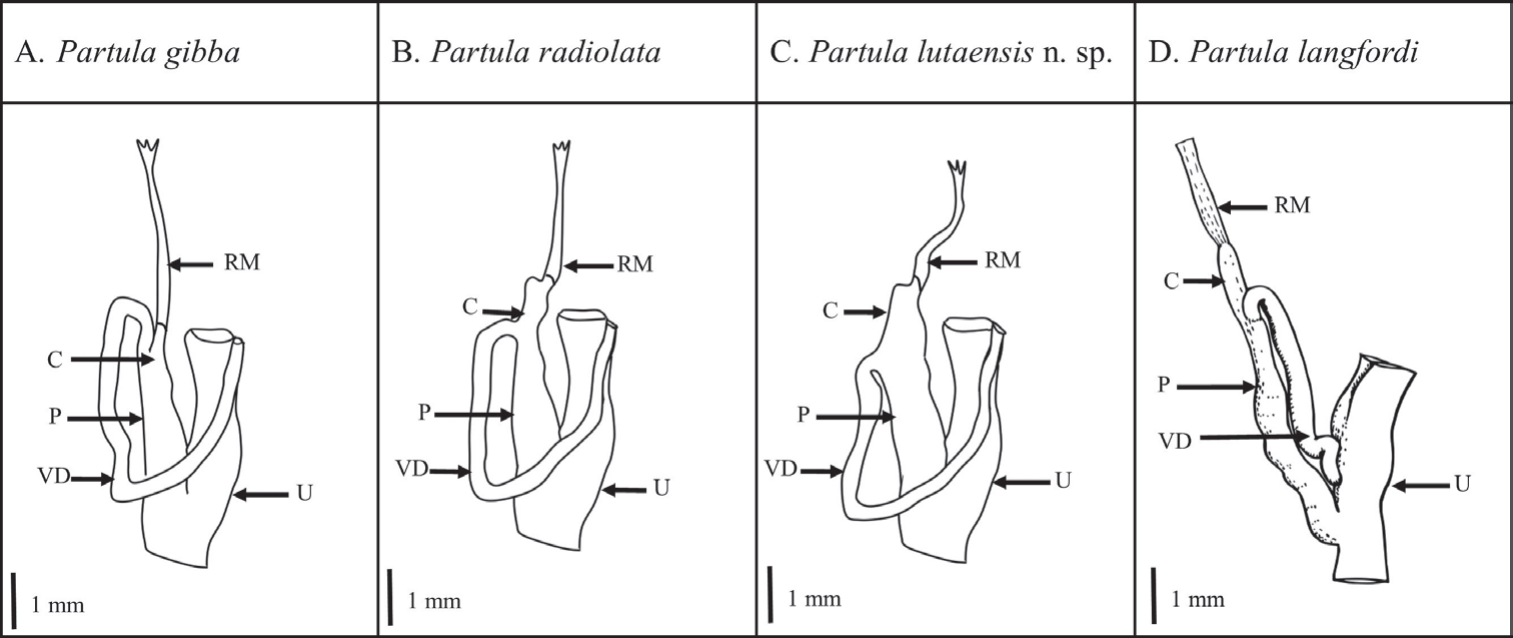
Outline drawings comparing the male reproductive tracks of four extant *Partula* species from the Mariana Islands. Male anatomy abbreviations are as follows: retractor muscle (RM), caecum (C), penis (P), vas deferens (VD), uterus (U). The figure of *Partula langfordi* is adapted from figure 5 of Kondo (1970). Note, Kondo found no difference between the male reproductive tracts of *P. gibba* and *P. langfordi*.

The male reproductive system of *Partula gibba* figured by Kondo (1955, 1970) and Gerlach (2016) is highly variable. In specimens we examined (Fig. 6A), the vas deferens entered the penis very near its top, leaving the upper portion of the penis, attached to the retractor muscle and called the caecum by Gerlach (2016), to be very short. In specimens of *P. radiolata*, the entry of the vas deferens was about 1/5 to 1/4 of the length of the penis below the retractor-muscle attachment (Fig. 6B). However, the male duct was distinctive in *P. lutaensis* sp. nov. by the bulge or shoulder at the top of an expanded caecum, proximal to the vas deferens. In the male system of *P. lutaensis* sp. nov., the attachment of the vas deferens was consistently more distal than in either *P. gibba* or *P. radiolata* (Fig. 6C); the insertion was close to 1/3 of the length of the penis below the retractor-muscle attachment. In no other regard were there any distinctive differences among the penial structures of these three species. The lower attachment of the vas deferens to the retractor muscle in both *P. lutaensis* sp. nov. and *P. radiolata* is concordant with their placement as sister taxa in phylogenetic reconstructions of the group. Kondo (1970) found no differences between the male reproductive tract of *P. langfordi* and *P. gibba*. We include a modified version of his drawing with ours for comparison (Fig. 6D).

###### Ecology.

Type and paratype specimens were found on *Epiprenmum aureum* and *Tectaria crenata* (J. Liske-Clark, Northern Mariana Department of Fish and Wildlife, personal communication).

###### Etymology.

The specific epithet *lutaensis* recognizes Luta, the indigenous Chamorro name for the island of Rota.

### DNA analyses

**Figure 7.**
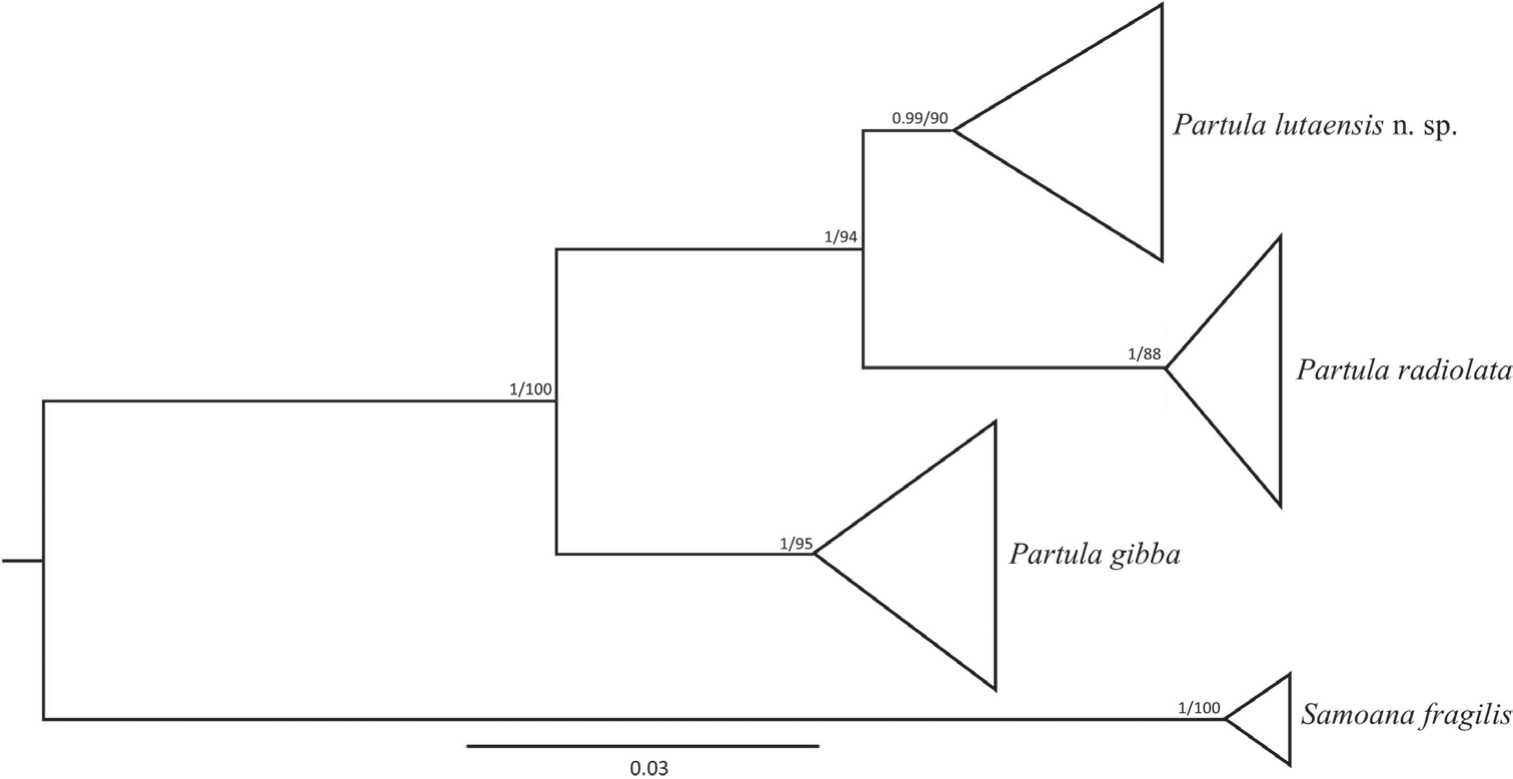
Bayesian phylogenetic tree representing the relationships between extant species in the family Partulidae from the Mariana Archipelago. This figure is an adaptation from one published in Sischo and Hadfield (2017). The phylogeny contains sequences from 24 individuals with unique haplotypes from seven islands and is based on a concatenated alignment of three genes (CO1, 16S and ITS2). The combined sequence length was 1683 base pairs. Maximum likelihood and Bayesian analyses recovered comparable topologies. Therefore, Bayesian posterior probabilities and maximum likelihood bootstrap values are reported on all nodes greater than 0.80 or 80% respectively. Branch ends have been collapsed to emphasize support for the species groups, rather than within group relationships. Also, note that this phylogeny does not include the newly sequenced haplotypes of *Partula lutaensis* sp. nov. mentioned above.

Analyses of the mitochondrial CO1 fragment confirmed that the tissue samples collected from the two type and paratype collection localities on Rota are *P. lutaensis* sp. nov. From the six tissue samples collected, two additional CO1 haplotypes were recovered (GenBank Accession numbers MT720839 and MT720840). As described in Sischo and Hadfield (2017), *P. lutaensis* sp. nov. is sister to *P. radiolata* from Guam despite having a shell more similar in appearance to *P. gibba* than to *P. radiolata* (Fig. 7). Currently all known extant colonies of *Partula* on Rota are *P. lutaensis* sp. nov.

## Discussion

The phylogeographic assessment of the extant partulids in the Mariana Islands reported by Sischo and Hadfield (2017) strongly supports the presence of a cryptic species on Rota and is concordant with further analyses of the male reproductive tracts, with *P. lutaensis* sp. nov. and *P. radiolata* sharing a lower attachment of the vas deferens in relation to the retractor muscle. Because this cryptic species was not found on any other islands, we conclude it is endemic to the island of Rota and have given it the name *Partula lutaensis* sp. nov. to recognize the indigenous Chamorro name for the island.

Available data indicate that all known populations of the genus *Partula* on Rota are *P. lutaensis* sp. nov.. This does not rule out the possibility that *P. gibba* once was, or currently is, on the island. Further surveys for extant partulid populations and analysis of sub-fossil shell remains on Rota may provide further evidence as to the present and historical distribution of these two species on the island. Moving forward, we strongly encourage that DNA barcoding be employed to determine species identification of any new living populations of *Partula* spp. discovered on Rota and elsewhere in the Mariana Islands. Furthermore, should *P. gibba* be located on Rota it should be attempted to find shell or body characters that might aid in distinguishing the two species without dissection or tissue sample collection.

*Partula lutaensis* sp. nov. was observed in locally high abundance on Rota, similar to observations of *P. radiolata* on Guam (Sischo and Hadfield 2017). Unfortunately, *P. gibba*, once the most abundant partulid on Guam, is now almost entirely extirpated (Hopper and Smith 1992; Sischo and Hadfield 2017; C. Fiedler personal communication June 2018). Persistence despite depredation by introduced predators may be further evidence of the shared ancestry between *P. lutaensis* sp. nov. and *P. radiolata*. Possibly, the *P. lutaensis* – *P. radiolata* clade shares behavioral and or life-history traits that have allowed the species to persist despite significant threats. For example, a recent study found that *P. radiolata* has a higher reproductive rate than *P. gibba* (Bick et al. 2018).

**Figure 8.**
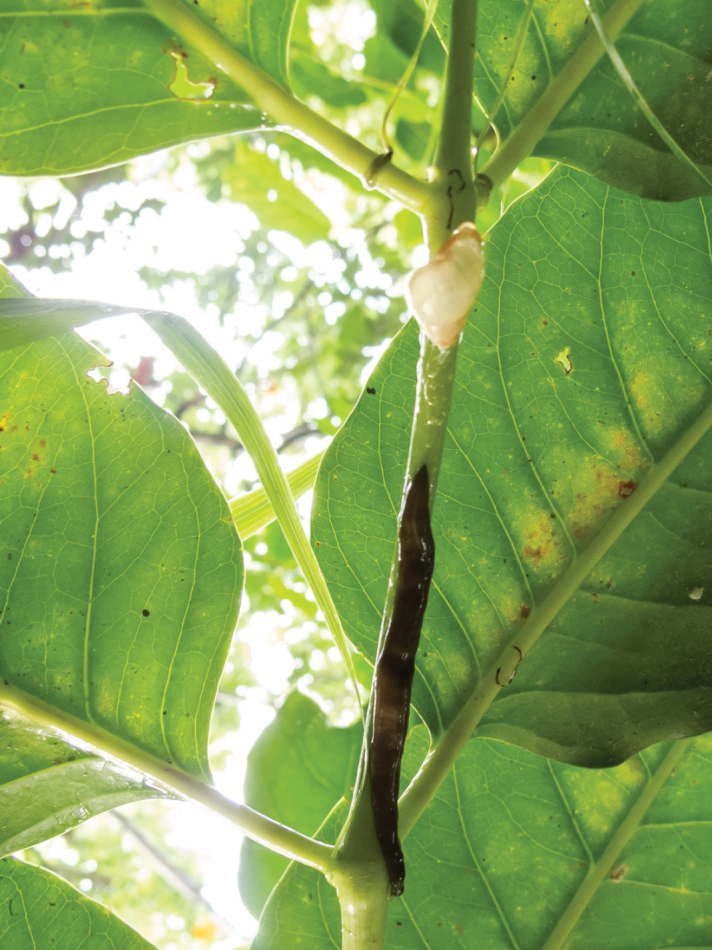
A *Partula lutaensis* sp. nov. freshly depredated by *Platydemus manokwari* observed by authors on Rota.

Across the Pacific, partulid species have been driven to extinction by introduced predators, most notably North American carnivorous snail species in the genus *Euglandina*, and the New Guinea flatworm *Platydemus manokwari* (Clarke, Murray and Johnson 1984; Murray et al. 1988; Hopper and Smith 1992; Bauman 1996; Coote et al. 1999; Cowie and Cook 2001; Régnier, Fontaine and Bouchet 2009; Pelep and Hadfield 2011; Meyer et al. 2017; Sischo and Hadfield 2017; Hadfield 2020; Gerlach et al. 2020). This has been particularly true in the Mariana Islands where half of the described partulid species are thought to be extinct, and the remaining species are imperiled across their ranges. While we observed *P. lutaensis* sp. nov. in locally high numbers in 2010, known populations are few and geographically discrete. Sub-fossil shells of partulids are ubiquitous on Rota (Bauman 1996) and Saipan (personal observations, October 2014), suggesting severe range reductions (Bauman 1996). Additionally, we observed *Platydemus manokwari* depredating *P. lutaensis* sp. nov. while collecting tissue samples on Rota (Fig. 8). Because *P. lutaensis* sp. nov. is an island-endemic species with a very restricted range and is clearly under significant predation pressure from introduced species, its existence is imperiled. When the Rota populations were considered to be *Partula gibba*, they were protected by a federal Endangered Species declaration. For these reasons, it is imperative that *Partula lutaensis* sp. nov. be listed as Endangered as soon as possible.

## Supplementary Material

XML Treatment for Partula

XML Treatment for Partula lutaensis
